# Less is more: new one-step intracameral chemotherapy technique

**DOI:** 10.22336/rjo.2021.44

**Published:** 2021

**Authors:** Davide Borroni, Chiara Bonzano, Rahul Rachwani-Anil, Carlos Rocha-de Lossada, Francisco Zamorano Martín, Maria Garcia-Lorente, Elisabetta Bonzano, Hussain Ahmad Khaqan

**Affiliations:** * Department of Doctoral Studies, Riga Stradins University, Riga, Latvia; ** Eye Clinic, DiNOGMI, University of Genoa and IRCCS San Martino Polyclinic Hospital, Genoa, Italy; *** Hospital Regional de Málaga-Hospital Civil; Department of Ophthalmology, Plaza del Hospital Civil, Málaga, Spain; **** Department of Radiation Oncology, Istituto di Ricovero e Cura a Carattere Scientifico (IRCCS) San Matteo Polyclinic Foundation, Pavia, Italy; ***** Department of Ophthalmology, Lahore General Hospital/ PGMI, Lahore

**Keywords:** intracameral, chemotherapy, retinoblastoma

## Abstract

**Purpose:** To describe the feasibility of a new one-step approach to aspirate the aqueous and apply melphalan in a single-go without repeated entries into the anterior chamber.

**Methods:** This retrospective non-comparative study was conducted at a referral center and included 12 patients. The one-step approach is described in a step-wise manner. No complications were observed among the patients.

**Results:** One single injection of intracameral melphalan proved to be a successful treatment in nine cases. Two patients required a second injection, which was administered two weeks after the first one following the same technique.

**Conclusions:** This proved to be a reasonable technique for the smooth application of melphalan in the anterior chamber studded with retinoblastoma seeds. Our outcomes revealed that it is an effective, quick, and cost-effective technique. Longer-term data collection is underway, though initial findings are encouraging.

## Introduction

Retinoblastoma (RB) is the most common ocular childhood malignancy [**1**]. RB has the highest survival rate, nearly 95% in the developed countries, compared to other pediatric intraocular malignancies [**2**,**3**].

Multidisciplinary care in RB management is mandatory. Treatment of RB is based on saving the patient’s life through a customized and risk-adapted approach to reduce systemic exposure to chemotherapy, optimize ocular drug administration, and safeguard vision [**4**]. In the management of intraocular RB, adequate chemoreduction and subsequent adjuvant treatment are usually planned instead of external beam radiotherapy (EBRT) [**5**,**6**]. For children affected by extraocular RB, systemic chemotherapy and radiation therapy (EBRT or plaque radiation therapy) are likely to be effective [**7**]. Otherwise, extraocular disease usually requires intensive systemic chemotherapy and may need additional high-dose myeloablative chemotherapy and blood-forming stem cell transplant with or without radiotherapy [**4**]. 

Enucleation is often necessary and is the treatment of choice in localized tumors, posterior surface of lens invasion, raised intraocular pressure (glaucoma) and buphthalmos [**7**]. Other indications for enucleation include the presence of pseudohypopyon, hyphema, iris nodules, vitreous hemorrhage impeding posterior segment examination, phthisis bulbi, and staphyloma [**7**]. Many attempts have been carried out to control the aqueous humor seeding, yet it still represents a real therapeutic challenge in RB management. Histopathology confirmation of anterior chamber involvement is also considered a criterion for enucleation, classified as stage E disease (International Classification of Retinoblastoma) [**8**]. The latter requires adjuvant chemotherapy [**1**]. 

Enucleation is hard to indicate in children, especially with only one functional eye [**9**]. However, it may be only avoided when globe-sparing therapies do not compromise the patient’s survival [**10**]. Recently, brachytherapy showed successful outcomes in the treatment of diffuse anterior RB not involving the retina [**11**,**12**]. Eye salvage through the combined use of intravitreal and intracameral melphalan is nowadays a successful technique and is being practiced [**9**]. Shields et al. reported that systemic chemotherapy and EBRT to control vitreous seeding showed a 5-year eye preservation rate of 47% and 53.4%, respectively [**10**]. Recently, a new intra-arterial chemotherapy technique has been developed, which showed a complete resolution of vitreous seedings in 67% of cases [**4**,**13**].

Nevertheless, diffuse RB infiltration to vitreous and anterior chamber that showed resistance to all available eye salvaging treatments inevitably leaves enucleation as the only option [**8**,**14**].

The ophthalmology department of Lahore General Hospital is one of the biggest referral centers for RB patients. In this study, we introduced a new technique of in situ chemotherapy, specifically developed to eradicate anterior chamber seeding (ACS) safely.

## Methods

This retrospective study was carried out in a hospital setting and included twelve diagnosed cases of RB with ACS. 

*Patient selection and surgical indications*

All patients underwent a careful examination under general anesthesia, with an accurate scleral depression to evaluate the entire retina, confirm the diagnosis of RB and define the correct location, extent of the tumor, and its staging. Complete retinal examination of both eyes and photographic documentation for future comparison were taken. 

Twelve patients, who were classified as group E, according to the International Classification of RB, were included in this study. All of them showed ACS. Intracameral injection of melphalan hydrochloride was performed to cause the regression of anterior chamber tumor seeding. A single surgeon managed all procedures.

*Surgical technique*

**Fig. 1:**
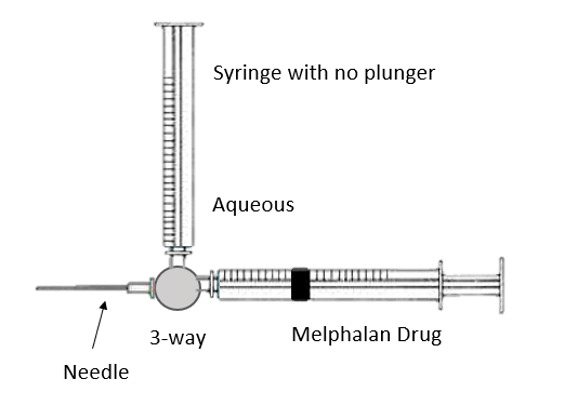
Three-way cannula attached to a 27-gauge needle

A three-way cannula attached to a 27-gauge needle was used in this study for intracameral injection of melphalan (**Fig. 1**). 

The distal end of the three-way cannula was attached to two syringes. One of the syringes had its plunger removed, creating a negative pressure in it. The negative pressure allowed aqueous humor to fill in this syringe once the three-way valve turned towards it. The other syringe contained 0.17 ml of melphalan to inject the drug into the anterior chamber and its plunger was in normal position. There is a new important concept of dead space in this technique. The area from the tip of the 27-gauge needle to the midst of the three-way cannula represented a dead space and its volume was 0.7 ml. Moreover, the distance from the midst of the three-way cannula to the tip of either syringe was 1 ml, so a total of 0.17 ml was a dead space and is highlighted in **Fig. 3** and **5**. 

**Fig. 2:**
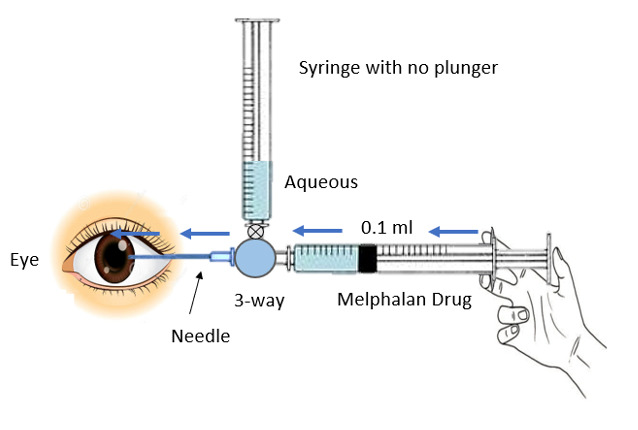
An adequate amount of aqueous is extracted

Once the patient was under general anesthesia, the surgeon held the three-way cannula with the attached 27-gauge needle and two attached syringes as shown in **Fig. 1**. After entering the anterior chamber with the 27-gauge needle under the microscope, the three-way port was turned towards the syringe with its plunger removed. Due to the negative pressure, aqueous humor was extracted. An adequate amount of aqueous was extracted, roughly 0.2 ml, although it was 0.37 ml due to 0.17 ml of dead space that went back into the eye, as shown in **Fig. 2**. 

**Fig. 3:**
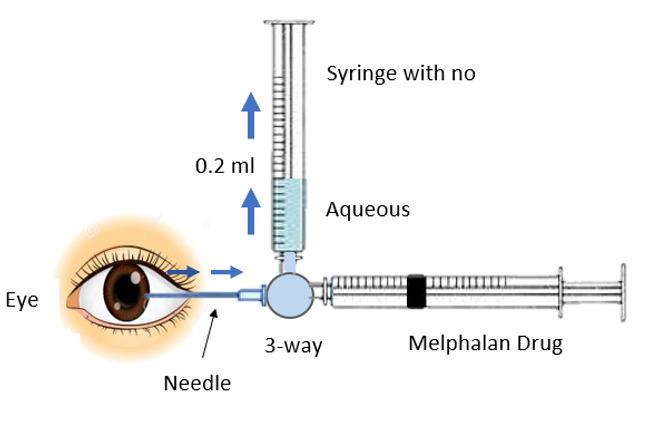
The area from the tip of the 27-gauge needle to the midst of the three-way cannula represented a dead space and its volume was 0.7 ml

**Fig. 4:**
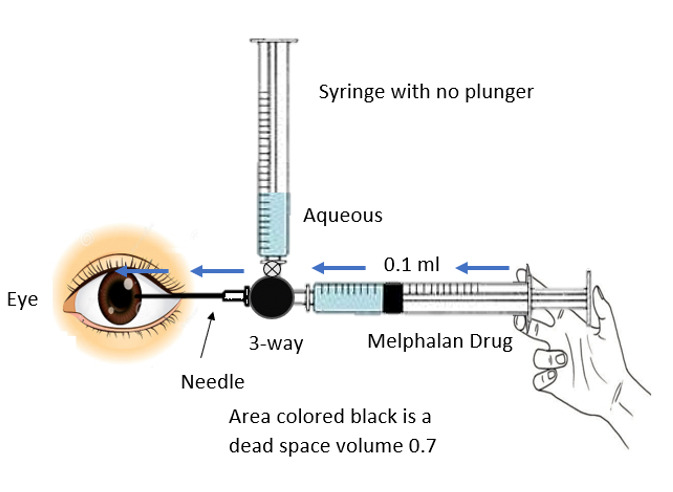
The three-way valve was then again rotated in the direction of the other syringe containing 0.17 ml of melphalan

**Fig. 5:**
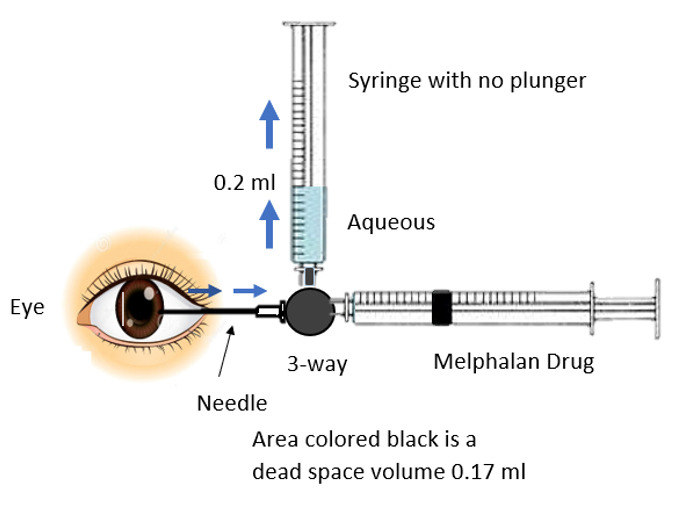
A total of 0.17 ml is the dead space

The three-way valve was then again rotated in the direction of other syringe containing 0.17 ml of melphalan, considering that 0.7 ml of drug remained stuck in the dead space once it was injected as shown in **Fig. 4** and **5**. Once the drug was injected, the needle was removed and cryotherapy was applied at the site of injection simultaneously, as well immediately after the removal of needle, in order to avoid the spillage of RB seeds from the site.

Proper consent was taken from all the patients and their attendants (parents) before taking their pre-injection and post-injection pictures.

## Results

One single injection of intracameral melphalan showed successful results in the treatment of nine cases with group E RB. Two patients required an additional injection, which was administered two weeks after the first one by using the same technique. One patient required three subsequent injections of intracameral melphalan for the complete resolution of ACS.

## Discussion

At present, a lot of work is being carried out and different techniques have been described, aiming at globe salvage treatments for patients with RB [**10**].

The first successful treatment procedure to control aqueous seeding was reported by Shields et al. [**15**]. They delivered iodine plaque therapy to the entire anterior segment, including the ciliary body, in three eyes affected by anterior diffuse RB without retinal or vitreous involvement. At 35-months follow-up examination, no recurrence occurred, and satisfactory visual acuity was preserved. It should be pointed out that radio-induced cataract, corneal limbal stem cell insufficiency and radiation-induced glaucoma could be long-term side effects [**8**]. Over the past few years, intravitreal chemotherapy has been developed in an attempt to reduce or complete elimination of vitreous tumoral seeding and to decrease the size of the lesion. When applied, intraocular chemotherapeutic drugs result in secondary salt and pepper changes at the site of lesion or in close proximity [**16**]. Further investigations include the combination of intravitreal chemotherapy and intraarterial chemotherapy together with transpupillary thermotherapy and brachytherapy [**17**]. Another chemotherapeutic drug, topotecan, displays an excellent rapid and potent antitumor effect in refractory or recurrent vitreous seeding [**18**-**20**].

The choice of local chemotherapy grants a higher effective dose at the tumoral site, allowing an effective treatment while reducing chemotherapy’s systemic side effects [**7**].

## Conclusion

We proposed a novel technique of local chemotherapy, consisting of the intracameral injection of melphalan in patients with a diagnosis of group E retinoblastoma with anterior chamber seeding. We have designed a special gadget and described the technique that allowed the administration of the drug in a safe, controlled, quick, and cost-effective manner. The main benefit of that gadget was that it allowed the surgeon to aspirate the aqueous humor and to inject melphalan in a single go without repeated entries into the anterior chamber, hence avoiding the risk of spillage of tumor cells. 

**Conflict of Interest statement**

The authors state no conflict of interest.

**Informed Consent and Human and Animal Rights statement**

Informed consent has been obtained from all individuals included in this study.

**Authorization for the use of human subjects**

Ethical approval: The research related to human use complies with all the relevant national regulations, institutional policies, is in accordance with the tenets of the Helsinki Declaration, and has been approved by the review board of Lahore General Hospital/ PGMI, Lahore.

**Acknowledgements**

None.

**Sources of Funding**

None.

**Disclosures**

None.
